# Supplementary material for: Complex‐centric proteome profiling by SEC‐SWATH‐MS
Source: Mol Syst Biol. 2019 Jan 14;15(1):e8438. doi: 10.15252/msb.20188438 (PMC6346213; doi:10.15252/msb.20188438)
Supplement: Supplementary file 7 — Dataset EV6 [file MSB-15-e8438-s007.zip › feature_plots_bioplex/P82912.pdf]

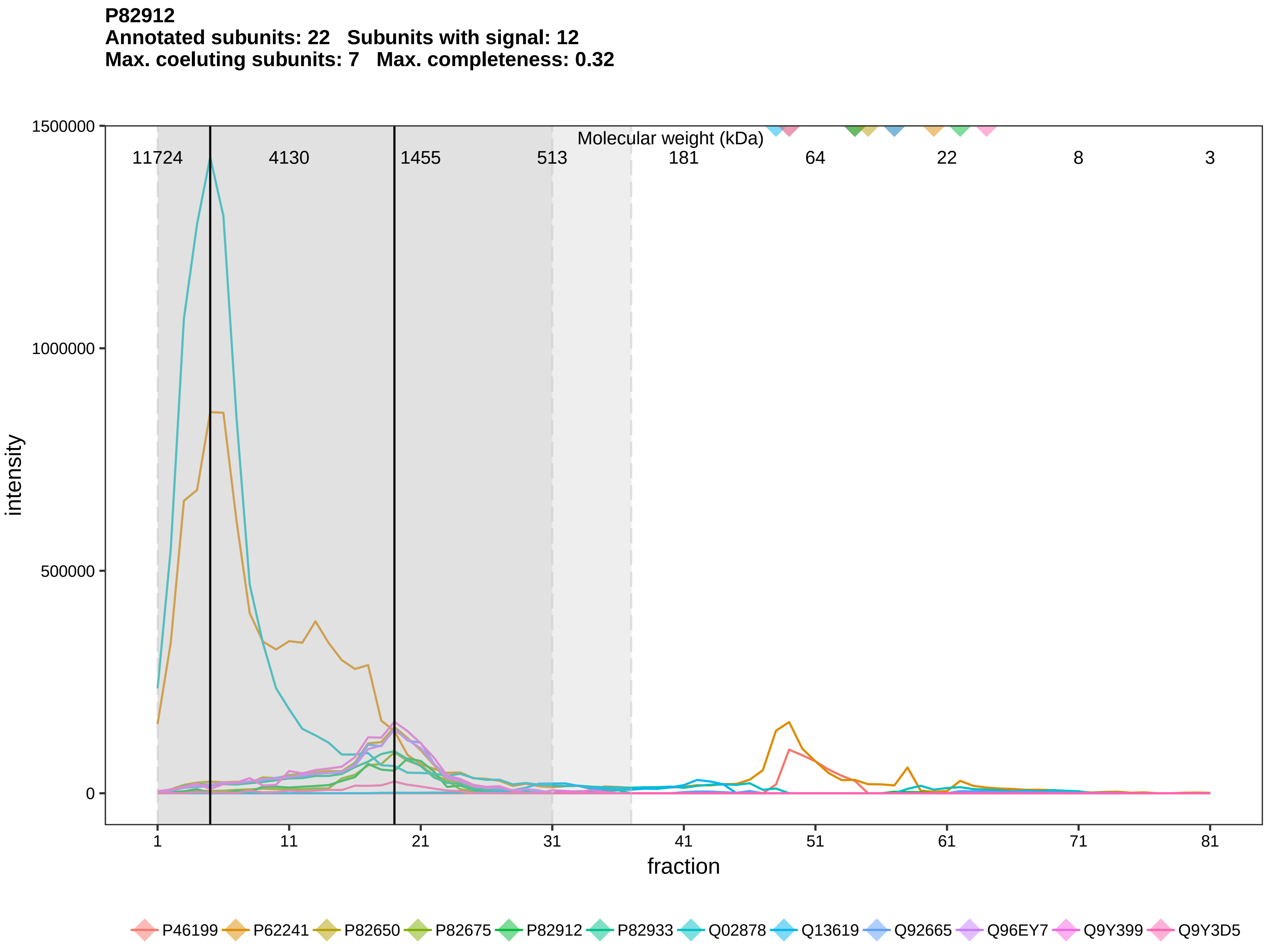
P82912
Annotated subunits: 22   Subunits with signal: 12
Max. coeluting subunits: 7   Max. completeness: 0.32
Molecular weight (kDa)
11724
4130
1455
513
181
64
22
8
3
intensity
1500000
1000000
500000
0
fraction
1
11
21
31
41
51
61
71
81
P46199
P62241
P82650
P82675
P82912
P82933
Q02878
Q13619
Q92665
Q96EY7
Q9Y399
Q9Y3D5
